# The Evolutionary Conserved Transmembrane BAX Inhibitor Motif (TMBIM) Containing Protein Family Members 5 and 6 Are Essential for the Development and Survival of *Drosophila melanogaster*

**DOI:** 10.3389/fcell.2021.666484

**Published:** 2021-09-03

**Authors:** Li Zhang, Sebastian Buhr, Aaron Voigt, Axel Methner

**Affiliations:** ^1^University Medical Center, Institute for Molecular Medicine, Johannes Gutenberg University Mainz, Mainz, Germany; ^2^Department of Neurology, RWTH Aachen University, Aachen, Germany; ^3^Forschungszentrum Jülich GmbH, JARA-Institute Molecular Neuroscience and Neuroimaging, RWTH Aachen University, Aachen, Germany

**Keywords:** calcium, mitochondria, ER stress, GRINA, lifeguard, MICS1, GHITM, Bi-1

## Abstract

The mammalian Transmembrane BAX Inhibitor Motif (TMBIM) protein family consists of six evolutionarily conserved hydrophobic proteins that affect programmed cell death and the regulation of intracellular calcium levels. The bacterial ortholog BsYetJ is a pH-dependent calcium channel. We here identified seven TMBIM family members in *Drosophila melanogaster* and describe their expression levels in diverse tissues and developmental stages. A phylogenetic analysis revealed that CG30379 represents the ortholog of human TMBIM4 although these two proteins are much less related than TMBIM5 (CG2076 and CG1287/Mics1) and TMBIM6 (CG7188/Bi-1) to their respective orthologs. For TMBIM1-3 the assignment is more dubious because the fly and the human proteins cluster together. We conducted a functional analysis based on expression levels and the availability of RNAi lines. This revealed that the ubiquitous knockdown of CG3798/Nmda1 and CG3814/Lfg had no effect on development while knockdown of CG2076/dTmbim5 resulted in death at the pupa stage and knockdown of CG7188/dTmbim6 in death at the embryonic stage. Ubiquitous knockdown of the second TMBIM5 paralog CG1287/Mics1 ensued in male sterility. Knockdown of dTmbim5 and 6 in muscle and neural tissue also greatly reduced lifespan through different mechanisms. Knockdown of the mitochondrial family member dTmbim5 resulted in reduced ATP production and a pro-apoptotic expression profile while knockdown of the ER protein dTmbim6 increased the ER calcium levels similar to findings in mammalian cells. Our data demonstrate that dTmbim5 and 6 are essential for fly development and survival but affect cell survival through different mechanisms.

## Background

Transmembrane BAX Inhibitor Motif (TMBIM) proteins are found in all of nature’s kingdoms, in bacteria, plants, and animals (Gamboa-Tuz et al., 2018). The name-giving human protein Bax inhibitor-1 (BI-1, TMBIM6) was originally identified in a yeast-based screen for its ability to inhibit cell death caused by overexpression of the pro-apoptotic protein Bax (Xu and Reed, 1998). All TMBIM proteins are characterized by the presence of multiple transmembrane spanning domains, share a specific signature (prosite PDOC00957) between the third and fourth transmembrane domain, and possess a semi-hydrophobic putative loop domain at their C-terminus. The six mammalian TMBIM proteins are localized at distinct intracellular membranes: TMBIM1 at the Golgi apparatus (Lisak et al., 2015) and lysosome (Zhao et al., 2006, 2017), TMBIM2 at the Golgi apparatus (Lisak et al., 2015), TMBIM3 at the Golgi apparatus and the endoplasmic reticulum (ER; Nielsen et al., 2011; Lisak et al., 2015), TMBIM4 at the Golgi apparatus (Gubser et al., 2007) and ER (Lisak et al., 2015), TMBIM5 at the inner mitochondrial membrane (Oka et al., 2008; Lisak et al., 2015), and TMBIM6 at the ER (Xu and Reed, 1998; Bultynck et al., 2012; Lisak et al., 2015).

Consistent with its subcellular localization, TMBIM6 affects the ER Ca^2+^ content; mouse embryo fibroblasts (MEFs) from BI-1^–/–^ mice showed an increased ER Ca^2+^ content (Chae et al., 2004), while over-expression of BI-1 in HT1080 cells (Chae et al., 2004) and in CHO cells (Westphalen et al., 2005) reduces it. This function is mediated by its C-terminal loop domain (Bultynck et al., 2012) and altered by pH (Kiviluoto et al., 2013; Bultynck et al., 2014). Recent structural and biochemical work with a bacterial TMBIM protein from Bacillus subtilis, BsYetJ, also confirmed a function as a pH-dependent ion channel. Crystal structures obtained in closed and open conformations were reversibly interconvertible by change of pH and recombinant BsYetJ reconstituted in proteoliposomes mediated Ca^2+^ influx in a pH-dependent manner (Chang et al., 2014). Also TMBIM3, first described as glutamate receptor ionotropic NMDA protein 1 (GRINA; Kumar et al., 1991), and TMBIM4 [better known as GAAP (Gubser et al., 2007)] have been reported to modulate the resting ER (Ca^2+^) and the release of ER Ca^2+^ (Rojas-Rivera et al., 2012; Carrara et al., 2015).

Other members of the TMBIM protein family in mammals are TMBIM1/RECS1 (responsive to centrifugal force and shear stress gene 1 protein) (Shukla et al., 2011), TMBIM2/LFG (lifeguard; Somia et al., 1999), and TMBIM5/GHITM (growth hormone-inducible transmembrane protein) also known as MICS1 (for mitochondrial morphology and cristae structure 1) (Oka et al., 2008). All family members play a role in the regulation of programmed cell death, specifically upon ER stress. TMBIM1 apparently interacts with the Fas ligand receptor CD95/Apo1 in the Golgi apparatus thus preventing its transport to the cell membrane (Shukla et al., 2011). Knockdown (KD) of TMBIM2 induces more active Caspase 8 (Hurtado de Mendoza et al., 2011). Overexpression of TMBIM3 restrains apoptosis specifically induced by ER stress (Rojas-Rivera et al., 2012). TMBIM4 over-expression prevents apoptosis induced by different kinds of stimuli – the proapoptotic Bax, staurosporine, doxorubicin, C2-ceramide, as well as TNFα and Fas ligand while its KD results in spontaneous apoptosis (Gubser et al., 2007). TMBIM5, a family member targeted to the mitochondrial inner membrane protein, maintains mitochondrial morphology and decreases the release of cytochrome c from mitochondria which indirectly links it to the induction of apoptosis (Oka et al., 2008) possibly by interfering with the mitochondrial protein synthesis machinery (Seitaj et al., 2020). Overexpression of TMBIM6 inhibits cell death caused by BAX, etoposide, staurosporine and growth factor withdrawal, possibly by forming a complex with the anti-apoptotic proteins BCL-2 and BCL-X_L_ (Xu and Reed, 1998). Knockout of TMBIM6 increases the susceptibility for ER stress (Chae et al., 2004) which was later attributed to its role as a direct inhibitor of the ER stress sensing protein inositol-requiring protein 1α (IRE1α; Lisbona et al., 2009), a serine/threonine protein kinase and endoribonuclease inserted in the ER membrane that represents the most ancient and conserved branch of the unfolded protein response (UPR).

TMBIM1 knockout (KO) mice are prone to cystic medial degeneration (Zhao et al., 2006) and accelerated metabolic cardiomyopathy caused by high fat diet-induced through activating proinflammatory factor cytokines such as the nuclear factor-κB (NF-κB; Gong et al., 2018). Cardiac-specific TMBIM1 KO causes a pathological cardiac hypertrophy by lack of lysosomal degradation of the activated proinflammatory and prohypertrophy factor Toll-like receptor 4 (TRL4; Deng et al., 2018), while TMBIM1 overexpression in hepatocytes of mice inhibited high-fat diet–induced insulin resistance, hepatic steatosis and inflammation by promoting lysosomal degradation of TRL4 (Zhao et al., 2017). TMBIM2 KO mice are viable but suffer from cerebellar atrophy caused by a reduced internal granular layer thickness and arrested Purkinje cell development correlating with increased levels of activated caspases 8 and 3 in the affected cells (Hurtado de Mendoza et al., 2011). Furthermore, cerebellar slices from TMBIM2 KO mice are more susceptible to Fas-mediated cell death (Hurtado de Mendoza et al., 2011) and more vulnerable to 1-methyl-4-phenyl-1,2,3,6-tetrahydropyridine (MPTP), an animal model of Parkinson’s disease (Komnig et al., 2016). TMBIM3 KD in the zebrafish results in embryonic lethality caused by disordered apoptosis (Rojas-Rivera et al., 2012). Using the *Drosophila melanogaster* model system, KD of a possible TMBIM3 ortholog or dTmbim6 alone resulted in a reduced eclosing rate only when exposed to tunicamycin whereas dmTMBIM3 and 6 double KD flies displayed a decreased eclosing rate *per se* which was even more pronounced upon treatment with tunicamycin (Rojas-Rivera et al., 2012). We have shown previously that TMBIM6 KO mice are obese and suffer from leukopenia and erythrocytosis, showing more splenic marginal zone B cells and nuclear translocation of NF-κB, changes that correlated with increased cytosolic and ER Ca^2+^ levels, but not with constitutive ER stress (Lisak et al., 2016; Philippaert et al., 2020).

So far, no conclusive, comparative analysis of the function of the TMBIM protein family in Ca^2+^ homeostasis and apoptosis has been conducted on an organismal level. In this study, we identified seven TMBIM family members in the fruit fly *Drosophila melanogaster* and compared their expression levels in different tissues and developmental stages. We then focused on strongly expressed family members and investigated the effect of ubiquitous and tissue-specific RNAi-mediated KD on development and lifespan and potential mechanisms of action. Our data demonstrate that TMBIM proteins are present in the fruit fly and that at least two family members are essential for fly development and survival but affect survival through different mechanisms despite their shared protein structure.

## Materials and Methods

### Phylogenetic Analysis

Multiple sequence alignment was performed using the online tool PRALINE using the Blosum62 scoring matrix with default settings (Henikoff and Henikoff, 1992). Alignments were preprocessed using PSI-BLAST with three iterations and an *E*-value cutoff of 0.01 (Simossis et al., 2005). The phylogenetic tree was constructed using the online tool phylogeny.fr (Dereeper et al., 2008). In short, the tool aligns the sequences using MUSCLE (3.7) with default settings (Edgar, 2004). After alignment, ambiguous regions were removed by Gblocks (v0.91b) and reconstructed in a phylogenetic tree using the maximum likelihood method in PhyML (v3.0 aLRT). Graphics were created using TreeDyn (v198.3). The multiple sequence alignment of TMBIMs across species was created using MAFFT version 6 using the E-INS-i setting and the alignment trimmed with JalView 2.5 at a cut-off value of 85% gaps. For the analysis of the secondary structure of the TMBIM family, the amino acid sequence of each TMBIM protein family member was entered as plain text into the “Prediction of Transmembrane Regions and Orientation” (TMpred) application (ExPASy) and the prediction graphic of the preferred model with the highest score was chosen.

### Fly Strains

Fly stocks were obtained from the Bloomington Stock Center (BDSC) and the Vienna Drosophila Resource Center (v): tubP-Gal4 (BDSC5138), elav-Gal4 (BDSC458), Mef2-Gal4, UAS-tdTomato/TM6B (donated by Olaf Vef), Pdf-Gal4 (BDSC6899), UAS-CG3798/Nmda1-RNAi (#1 v28365, #2 BDSC28361), UAS-CG3814/Lfg-RNAi (#1 v4671, #2 BDSC33354), UAS-CG2076-RNAi (#1 v50221, #2 BDSC64564), UAS-CG1287-RNAi (#1 v12127), UAS-CG7188-RNAi (#1 v3235, #2 v37108), UAS-Always early-RNAi as RNAi control (v13673), UAS-erGAP3 (donated by M. Teresa Alonso), UAS-4mtGCaMP3, UAS-RFP (donated by Ronald Davis). The targeting sequence of RNAi strains are listed in Table 1. Flies were raised at 25°C and fed with standard food if not otherwise stated.

**TABLE 1:** List of targeting sequence of RNAi strains.

RNAi strain	Targeting sequence
UAS-CG3798/Nmda1 #1 v28365	TCTATGGCTTGCTGCGAAAGTGTGCGCCGCCAAACACCGACGAACTTCATATTCTTGGGTCTGTTTACAGCAGCTCAGTCG TTCTTAATGGGAGTTTCCGCAACCAAATATGCTCCGAAAGAGGTTCTCATGGCGGTGGGCATAACAGCAGCGGTTTGCTTGG CCCTAACGATCTTCGCCTTGCAGACAAAGTACGACTTTACTATGATGGGCGGTATTTTGATCGCCTGCATGGTGGTGTTCCTG ATCTTCGGCATCGTGGCCATCTTTGTAAAGGGAAAGATCATAACACTGGTGTACGCCTCGATTGGAGCGCTGCTCTTCTCC GTTTATCTCATCTACGACACACAGTTAATGATGGGCG
UAS-CG3798/Nmda1 #2 BDSC28361	CACCCTATGCACAAGGAGGTGCTCAACCCTATCCACAGCCCTACGGACAGGGGCCTCCACCGGGAGGTTATGCTCCCCA GCCGGGATTTATCCAACCACCACCATCTGCTGGCGGCTACGGAGCCTACGATGATCCGGAGAGCCAGCCCAAGAACTTC TCGTTTGACGACCAAAGCATCCGTCGCGGATTCATACGTAAGGTGTACCTGATTCTAATGGGACAACTAATCGTCACTTTT GGAGCTGTTGCCCTGTTTGTATACCACGAGGGCACTAAAACCTTTGCCAGGAATAACATGTGGCTCTTTTGGGTTGCCCTC GGCGTAATGTTAGTAACCATGCTGTCTATGGCTTGCTGCGAAAGTGTGCGCCGCCAAACACCGACGAACTTCATATTCTTG GGTCTGTTTACAGCAGCTCAGTCGTTCTTAATGGGAGTTTCCGCAACCAAATA
UAS-CG3814/Lfg #1 v4671	CGGACAGTTTGAGGCTGATGAGGTCCTGATGGCAGTGGGAATTACGGCCGCGGTGGCCCTGGGACTCACCCTGTTTGC CCTGCAGACCAAATACGATTTTACGATGTGCGGAGGAGTGTTGGTGGCCTGTCTGGTGGTCTTCATCATTTTTGGTATA ATCGCTATCTTCATACCGGGCAAGGTGATCGGACTGGTCTATGCCTCCTTGGGAGCATTGCTCTTCTCCGTTTACTTGGTGTA CGATACCCAGTTGATGCTGGGTGGTAATCATAAGTACTCCATCAGTCCTGAGGAATACATCTTCGCCGCTCTAAACCTCTACC
UAS-CG3814/Lfg #2 BDSC33354	TTGGATTAGTTAGTCGTCTTG
UAS-CG2076 #1 v50221	CCGTGAGCCCGTCGAAGAGATGCGTGCTCCCTCGCTGAAGGAGAAACTGATGGGTCCGCCAAGTGCCAATGCATACTC CATGGGCAAGGGCGCTGCCGCCGGAGCAGCCGCCGTGGGATTGGGCGCCCTGTGCTACTACGGCGTGGGATTGGGA AAACAAACCAGCATCGCGGATAATGCCATCATGTGGCCCCAGTTTGTGCGCGATCGTATCCAGAGCACCTATGCCTT TTTCGGTGGCTCTTGCGTTCTCACCGCGGCCGCAGCAGCAGCCACCTTCCGTTCGCACCGTCTCCTGGAGCTGGCCT CGCGTGGCGGCATCTTGGCGACCATTGCTTCGCTGGCGCTGGTCATT
UAS-CG2076 #2 BDSC64564	ATGTAGAGGAACTTATCGCTG
UAS-CG1287 v12127	GCCAATCAACCGAGTATCTACGACCACTCGATGGTGTGGC CCCAGTACGTGAGGGATCGTATTCATGCCACCTATGCCTA TTTCGGAGCATCCTGTGGTGTAACAGCTGCTTCAGCCGTTGCCTTTTTCCAATCGGATGCCATGATGGCTCTGATGAC GCGTTCTGGATGGGTTGCCTCATTGGTTACCCTGGGACTGGTAATGCTCAGTGGATCCATTGCACAAGGCCTAGAATA TCAGCCGGGATTCGGAGCCAAGCAGCTGGCCTGGCTCGTCCACTGTGCCGTTCTGGGAGCTGTC
UAS-CG7188 #1 v3235 #2 v37108	GCGCGAGCACCTGTCTAAGGTTTACATGGTCCTGGGCAGCACTGCCGCTGCCACGGCCATGGGAGCCATGCTTCA GATGCGTGACTTTCTCGATCTTGGAGTCCTGGCGGCGGTGGCCACTCTAGTCCTGGTCTTGGGTCTGCACTTCTA CAAGGATGACGGCAAGAACTATTATACACGTTTGGGCATGCTCTACGCCTTCGGATTCTGCTCCGGCCAGACGCTCG GACCGCTCCTCGGCTATATATGCAGCATAAATCCGGCAATAATCCTGTCTGCCCTTACGGGCACCTTCGTCACCTTC ATCTCGCTCTCCT TGTCCGCCCTTCTGG
UAS-Always early v13673	CCGTTGGATTTGGTGTGAGTTCGTCGACTCCTTCCTGGACAAGCCGACCCTGACCATGGGCTACGATATGAAGCGCTTC ATAGCGGAGTACTGTCCGCTCCTGCACTCTTGCTTCATGCCCCGCAGAGGATGGCAATTGGTACGTCGGAATATGGG GAAGGCGCGTCGATTTTCGGCCGCCTTCATCGAGCTGGAACGCGAAGAATTGGAGTGCCAGCGCCGCATTGTGCGCC AGTTGCAGCAGCATAAGTTCAATCCCAAGGAGAACGTGGGCTACTTGGACCAGATACCCAAGCGTGTGCCCCTGCCA CTGGCCAAGGATGCCACGGTCAGCA

### Analysis of Development and Behavior

Aging: Flies were transferred to fresh food and the number of dead flies was recorded every 3 days. In some experiments, 10 μM rotenone or tunicamycin dissolved in 5% sucrose solution was added to filter papers in the fly vial. Fresh solution was added twice a day to make sure that the filter paper was wet but not flooded. The number of dead flies was noted everyday. 5% sucrose alone served as control. Eclosion: Pupa eclosing rate was observed until the (n−1) × 2 day (n is the day of first F1 eclosing) after crossing. For Mef2-Gal4 KD flies, the parents of the cross were Mef2-Gal4, UAS-tdTomato/TM6B virgin flies and UAS-RNAi males. Therefore, the normal distribution of offspring should be 50% KD flies and 50% non-KD flies. The red fluorescent signal from tdTomato allows distinguishing KD pupae and non-KD pupae. We marked the pupae on the vial to monitor whether the pupae eclosed. For elav-Gal4 KD flies, since the parents were homozygous, the number of empty pupae and black pupae per cross were noted at the designated day and the percentage of successful eclosion (number of empty pupae divided by the number of total pupae) was calculated. Each cross, roughly yielding 50–200 offspring, was regarded as one group. Wing phenotype: The number of flies with abnormal wings was scored on day 2. Each group of offspring from one crossing has 50–200 flies, and at least three groups/crosses per genotype were counted. Crawling: The speed of larvae crawling was determined by the distance covered within 1 min after centering the larva. Climbing: The speed of adult climbing was analyzed by video recording of flies in a 15 ml Falcon tube after being tapped down on the bench. Each tube contained 10 flies and the video was recorded for 15 s after the tapping. Five groups per genotype per age were examined. All assays were conducted at the same time of day and all flies should be mated because the probe contained male and female flies. UAS-Always-early-RNAi was used as a control RNAi group except for male fertility tests. Always early encodes a protein expressed in testis where it controls the onset of spermatid differentiation and G2-meiosis I transition (White-Cooper et al., 2000). Male fertility analysis: Virgins of w/Y; tubP-Gal4/TM3, Sb were crossed to males w/Y; CG1287-RNAi. In the F1 generation, we selected males with either ubiquitous CG1287 KD (w/Y; CG1287-RNAi/tubP-Gal4) or without CG1287 KD (w/Y; CG1287-RNAi/TM3, Sb) as controls. 10 males of each genotype were individually crossed to 3–5 virgins (yw; Sco/CyO). The absence of the filial generation was used as an indicator for male sterility.

### ATP Quantification

Adenosine triphosphate (ATP) was measured in whole L3 larvae or pupae (avoiding black pupae) as described in Liu and Lu (2010) Briefly, two larvae or pupae were lysed in 100 μl lysis buffer from ATP Bioluminescence Assay Kit HS II (Merck 11699709001), heated at 95°C for 2 min, and then centrifuged at maximal speed at 4°C for 1 min. 2.5 μl clear supernatant, 187.5 μl dilution buffer, and 10 μl luciferase from the kit were mixed and the luminescence was immediately measured using a Spark Multimode Microplate Reader from Tecan. The absolute ATP amount was calculated according to an ATP standard curve.

### Microscopy

To monitor mitochondrial calcium and ER calcium, brains of pdf > > 4mtGCaMP3, RFP and pdf > erGAP3 adult flies were dissected and prepared as described in Manning et al. (2017) and imaged by Leica SP2 with laser intensity 20%, Alexa 488 and RFP, Gain 20%. Alexa 488 indicates calcium and RFP marks the nucleus. Regions of interest were drawn around Pdf neurons and the mean intensity measured by NIH ImageJ. For mitochondrial calcium, the mean intensity of the experimental sample was normalized to the mean value of the control genotype, as well as to the mean intensity of RFP signal from the same neuron.

### Quantitative PCR

Total RNA was extracted using the ZR RNA MiniPrep kit (ZYMO RESEARCH) and cDNA synthesized from 10 ng/μl RNA using the High Capacity cDNA Reverse Transcription Kits (Life Tech). Quantitative PCR (qPCR) was conducted by Takyon No Rox Probe Master Mix dTTP (Eurogentec) with 2 μl of cDNA and a final concentration of 570 nM Primer and 100 nM Probe. The primers were from Eurofin and probes from the Universal Probe Library (Roche) and are listed in Table 2. The transcriptional levels were calculated by the 2^–ΔΔCt^ (Ct, cycle of threshold) method. ΔΔCt = ΔCt of experimental group – mean ΔCt of control groups. ΔCt = Ct (gene of interest)−Ct (housekeeping).

**TABLE 2:** List of probes and primers.

Gene	Probes and primers
CG3798/Nmda1	#131, caccgagctgctgtgtatgt, cgaaaccgctgtggagtaa
CG3814/Lfg	#40, cggccacctgttcagtagtt, gtgtatgcgccagtttcatc
CG2076	#57, aaccagcatcgcggataat, aaaaggcataggtgctctgg
CG1287/Mics1	#57, gtctgtgctactatggtctgggc, ggcataggtggcatgaatacgat
CG7188/BI1	#36, gctgattatactgacgcaaaagc, ggataatctgattgagctattttgg
Rp49/CG7939	#117, cggatcgatatgctaagctgt, gcgcttgttcgatccgta
spliced Xbp1/CG9415	#116, gatctgccgcagggtatacaac, ggccacaactttccagagtg
non-spliced Xbp1/CG9415	#89, ctgtgcgtccaccaacct, tgtgtccacctgttgtataccc
Buffy/CG8238	#148, ccgtggattaacgagaatgg, caggctgttggtagtgggtaa
Debcl/CG33134	#51, caggagtacaaaatggatatcatca, gtgtcaccttccggttgag
Diap1/CG12284	#5, gatgtggctccgtccgta, cgcctcgacgatcttgtt
Diap2/CG8293	#7, gaagtgtgtttggtgcaacg, gctcttcaaaggcattgtcg
Bruce/CG6303	#4, gcctgaggagaaatggaatg, gatcagggactggatggaaa
Det/CG12265	#2, gccaggactccctatcgtatg, agtggatacagcggatgttgt
p53/CG33336-A	#76, tgatcagatatagccgactaagatgta, tcatcctcggaatcagtgct

### Statistical Analysis

The Shapiro–Wilk test was used to determine normality of distribution of the data. Data normally distributed were analyzed with the one-way ANOVA followed by the Dunnett’s or Šídák’s multiple comparison test, two-way ANOVA followed by Tukey’s test or with the independent *t* test. Data not-normally distributed were analyzed with the Kruskal–Wallis test followed by the Dunn’s test or with the Mann–Whitney test. The log-rank test and the Gehan–Breslow–Wilcoxon test were used to assess differences in lifespan. It was considered to be statistically significant when both tests showed significant differences. A *p* value lower than or equal to 0.05 was considered statistically significant.

## Results

### Identification of TMBIM Proteins in the *Drosophila melanogaster* Genome

**FIGURE 1:**
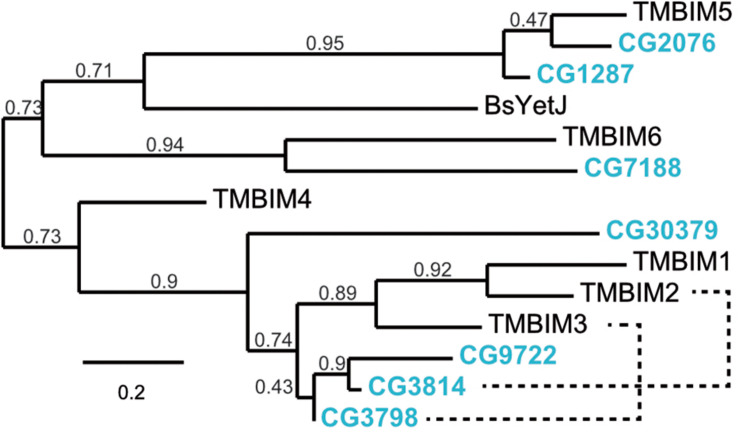
Phylogenetic relationship of fly and human TMBIM proteins. Phylogenetic analysis of the sequence alignment of human and Drosophila TMBIM proteins identifies CG2076 and CG1287/Mics1 as orthologs for TMBIM5 and CG7188/BI-1 for TMBIM6, TMBIM4 is most probably CG30379 while the relationship of TMBIM1-3 to their fly orthologs is less clear. The bacterial homologue BsYetJ is most closely related to TMBIM5 and TMBIM6. The branch support values are indicated above the branches. The phylogenetic tree was created using the Maximum Likelihood algorithm with the BLOSUM62 substitution matrix and 100 bootstrap trials. The dashed lines indicate closest homologues according to BLASTP.

To establish Drosophila as a model in which to study organismal TMBIM function across the protein family, we first used the BLASTP algorithm^1^ with human TMBIM1-6 as input which identified seven putative fly TMBIM proteins in the *Drosophila melanogaster* genome. We then constructed a phylogenetic tree of all fly and human TMBIM proteins using the bacterial homologue BsYetJ as outlier (Figure 1). From this analysis it appears that the fly TMBIM proteins fall into two distinct groups consisting of proteins homologous to TMBIM1-3 and TMBIM4-6 similar to the vertebrate TMBIM proteins (Lisak et al., 2015). The fly proteins CG2076 and CG1287/Mics1 are both potential orthologs of human TMBIM5. Human TMBIM5 consists of 345 amino acids (aa), CG2076 of 341 aa and CG1287/Mics1 of 365 aa. The two fly proteins are 59% identical and 75% similar to each other. CG2076 and human TMBIM5 are 48% identical and 64% similar, the alignment produces 6% gaps. CG1287 and human TMBIM5 are 47% identical, 63% similar, and the alignment has 8% gaps. This slightly closer relationship of CG2076 with human TMBIM5 is also evident in the phylogenetic analysis (Figure 1). CG7188/BI-1 appears to be the ortholog of human TMBIM6. CG30379 probably represents the ortholog of human TMBIM4 although these two proteins are much less related than TMBIM5 and 6 with their respective orthologs. The relationships between fly CG3798/Nmad1, CG3814/Lfg, CG9722, and human TMBIM1-3 are more dubious as all three fly proteins and all human proteins are more closely related to each other than to their putative orthologs. In an effort to match fly and human orthologs, we therefore added serial BLASTP of two sequences to the analysis which suggested that human TMBIM2 corresponds most likely to CG3814/Lfg and human TMBIM3 to CG3798/Nmda1 leaving CG9722 as the most probable human TMBIM1 ortholog. This grouping is also supported by the relative relationships between the proteins in their respective branches where human TMBIM1 is more closely related to human TMBIM2 similar to fly CG9722 being more closely related to fly CG3814/Lfg. We conclude that a definite attribution of the fly proteins to their human counterparts is not useful for the branch consisting of CG3798/Nmda1, CG3814/Lfg, and CG9722 (TMBIM1-3), as these proteins are much more closely related to each other than to the human proteins and probably evolved from a common ancestor. Such an attribution is, however, obvious for the other branch of TMBIM4-6 orthologs. CG2076 is actually closer related to the human TMBIM5 protein than its paralog CG1287. We will therefore henceforth use dTmbim5 for CG2076 and dTmbim6 for CG7188 while the remaining proteins will be addressed by their fly names.

### Expression Pattern of Fly TMBIM Proteins

**FIGURE 2:**
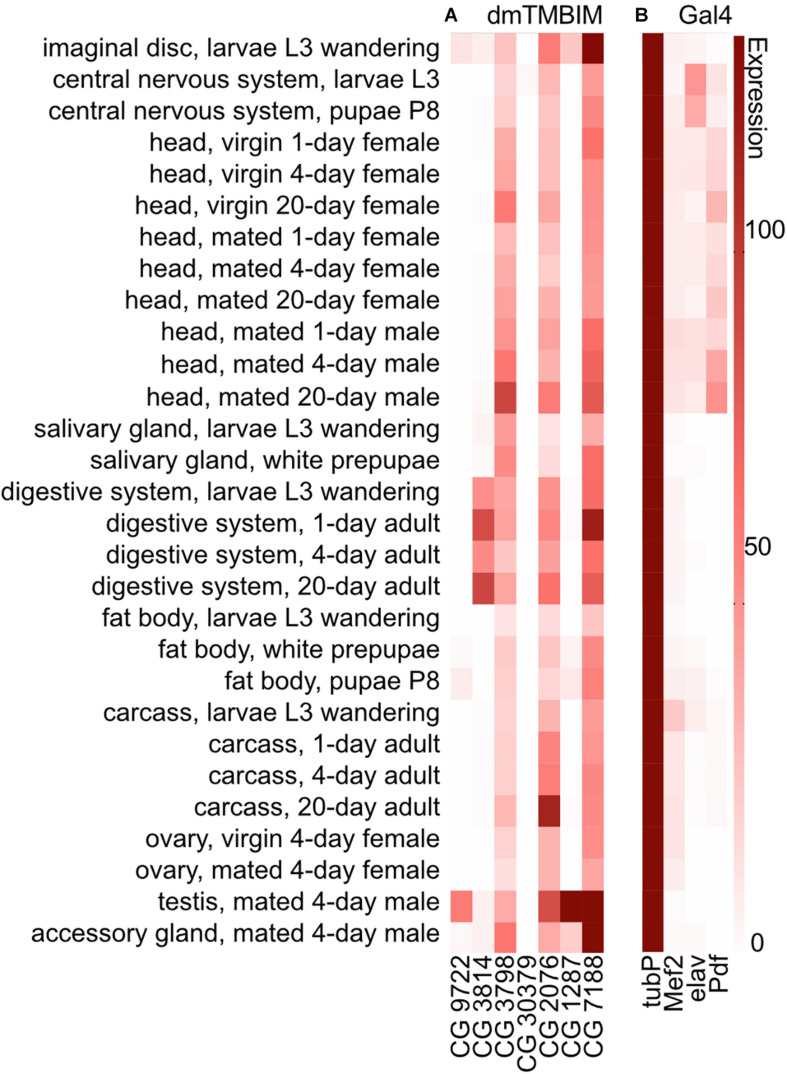
Expression of mRNAs encoding fly TMBIM proteins and Gal4 drivers used in this study. **(A)** The expression of mRNAs of fly TMBIM proteins (dmTMBIM): CG9722, CG3814/Lfg, CG3798/Nmda1, CG30379, CG2076/dTmbim5, CG1287/Mics1, and CG7188/dTmbim6/BI-1. **(B)** The expression of Gal4 proteins used in this study. 0: no/extremely low expression; 0–10: very low/low expression; 11–25: moderate expression; 26–50: moderately high expression; 51–100: high expression; over 100: very high expression.

To clarify the expression of the fly TMBIM family members in a comparative manner, we compared their temporal and tissue-specific expression using published modENCODE mRNA-Seq data (Brown et al., 2014; Figure 2A). During development, CG9722 is detectable in the imaginal disk of L3 larvae and the fat body of P8 pupae. In adults, it is essentially only expressed in the testes. CG3814/Lfg is expressed mainly in the imaginal disk and salivary gland of L3 wandering larvae and in the adult digestive system. CG3798/Nmda1 is ubiquitously expressed with the strongest expression in adult heads. CG30379 (putative dTmbim4) is expressed only in very low amounts in the central nervous system from late L3 larva to the early pupa stage. Regarding the orthologs of TMBIM5, CG1287/Mics1 is expressed strongly in the testes, the accessory glands of males and in the imaginal disk of L3 wandering larvae while dTmbim5 is expressed strongly and almost ubiquitously at all developmental stages. In adult flies, the highest expression levels are found in the carcass and the testes. This further supports that dTmbim5 is the ortholog of TMBIM5 which is also almost ubiquitously expressed (Reimers et al., 2007; Oka et al., 2008). dTmbim6 is similarly expressed in all tissues and at all developmental stages. In adult flies, it is most strongly expressed in the testes and the accessory glands.

### CG3798/Nmda1 and CG3814/Lfg Are Not Essential for the Development of *Drosophila melanogaster*

Based on these data and the availability of suitable RNAi lines, we decided to focus on the four family members with the strongest and most ubiquitous expression, CG3814/Lfg, CG3798/Nmda1, dTmbim5, and dTmbim6. CG1287/Mics1 was included to clarify which of the two TMBIM5 orthologs is more important for the correct development and wellbeing of flies. To knockdown (KD) these genes in different tissues, we used tubP (α-Tubulin at 84B) for ubiquitous expression, the somatic muscular Mef2 (myocyte enhancer factor 2), and the pan-neural elav (embryonic lethal abnormal vision) promoters.

**FIGURE 3:**
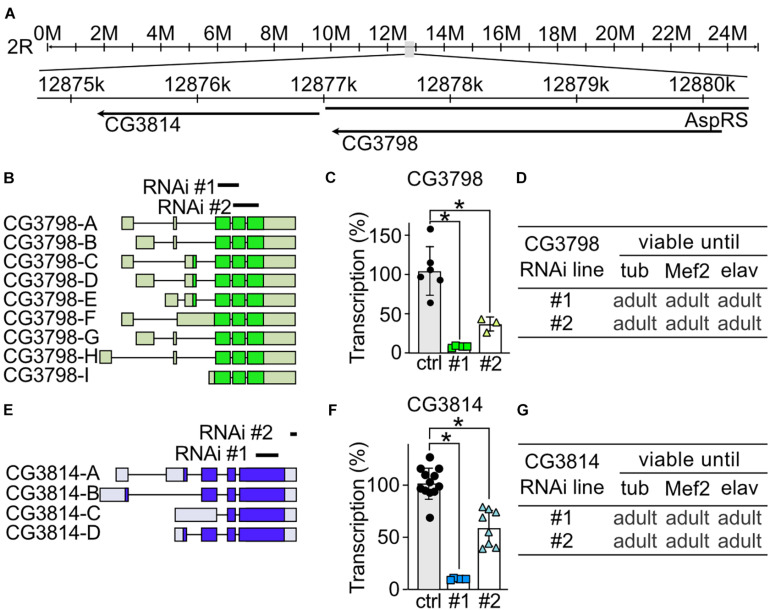
CG3798/Nmda1 and CG3814/Lfg are not essential for the development of *Drosophila melanogaster*. **(A)** Genomic location of CG3798/Nmda1 and CG3814/Lfg. M, million base pairs (bp); k, thousand bp; AspRS, Aspartyl-tRNA synthetase. **(B,E)** Splice isoforms of CG3798/Nmda1 and CG3814/Lfg, respectively. Dark color indicates the open reading frame. The targeting position of the two RNAi lines used in this study are marked as black lines. **(C,F)** Transcriptional levels of CG3798/Nmda1 and CG3814/Lfg in tubP > > CG3798/Nmda1-RNAi and tubP > > CG3814/Lfg-RNAi whole 2-day-old adult flies determined by qPCR. Each bar represents the mean ± SD and each dot a group of 10 flies. Statistical significance was calculated using one-way ANOVA followed by the Dunnett’s test. ns, not significant. *p < 0.05. **(D,G)** Viability of CG3798/Nmda1 or CG3814/Lfg KD flies by two RNAi lines driven by the indicated Gal4 drivers.

To study the role of CG3798/Nmda1 and CG3814/Lfg (both highly homologous members of the TMBIM1-3 cluster) in development, locomotion and lifespan, we assessed the phenotype of larvae and adult flies after knockdown with two independent RNAi lines. Notably, CG3798/Nmda1 localizes directly upstream of CG3814/Lfg in the genome suggesting a gene duplication event (Figure 3A). For CG3798/Nmda1, RNAi #1 targets the third and fourth exon and RNAi #2 the last two exons according to transcript A (Figure 3B). Both RNAi lines significantly reduced mRNA levels of CG3798/Nmda1 in whole adult *tubP* > > *CG3798/Nmda1-RNAi* flies (Figure 3C) but KD using ubiquitous, muscular and neural drivers had no effect on development, all flies eclosed into adult flies (Figure 3D). For CG3814/Lfg, RNAi #1 targets the last exon, while RNAi #2 3′ untranslated regions (UTR; Figure 3E). Both lines significantly reduced CG3814/Lfg mRNA as shown by qPCR studied in whole adult tubP > > CG3814/Lfg-RNAi flies (Figure 3F) but KD with all three drivers had no detrimental effect on the development of flies (Figure 3G). No obvious phenotype became apparent in both lines, CG3798/Nmda1 and CG3814/Lfg. Together, these results suggest that CG3798/Nmda1 and CG3814/Lfg are dispensable for fly development.

### Ubiquitous Knockdown of the Putative TMBIM5 Orthologs Results in Male Sterility for CG1287/Mics1 and Arrested Development at the Pupa Stage for dTmbim5

**FIGURE 4:**
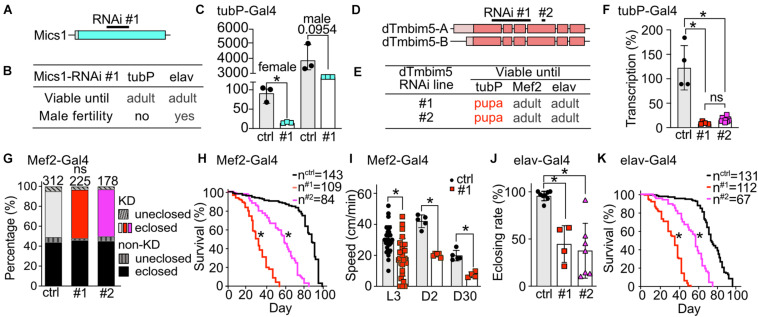
Ubiquitous knockdown of the putative TMBIM5 orthologues results in male sterility for CG1287/Mics1 and arrested development at the pupa stage for dTmbim5. **(A)** Genomic organization of CG1287/Mics1 which is encoded by a single unspliced exon. Lighter color represents the UTR and the darker color the open reading frame. The targeting sequence of the RNAi line is indicated. **(B)** Viability and male fertility of CG1287/Mics1 KD flies by RNAi driven by tubP- and elav-Gal4 at 29°C. **(C)** Transcriptional levels of CG1287/Mics1 quantified by qPCR from adult female or male tubP > > Mics1-RNAi flies raised at 29°C. **(D)** Splicing isoforms of dTmbim5. Lighter color represents the UTR and the darker color the open reading frame. The targeting sequence of the two RNAi lines is indicated. **(E)** Viability of dTmbim5 KD flies by two RNAi lines and various Gal4 drivers. Red means arrested development. **(F)** KD efficiency of UAS-dTmbim5-RNAis (#1, #2) assessed by qPCR from whole tubP > > dTmbim5-RNAi L3 larvae. **(G)** Eclosion and **(H)** lifespan of Mef2 > > dTmbim5-RNAi flies. **(I)** Locomotor activity of Mef2 > > dTmbim5-RNAi #1 larvae and flies. L3, L3 wandering larva; D2, 2-day-old; D30, 30-day-old adult flies. **(J)** Successful eclosion and **(K)** lifespan of elav > > dTmbim5-RNAi flies. Ctrl in **(C)** are UAS-vasa-RNAi flies and UAS-Always-early-RNAi in **(F–K)**. Bars in **(C,F,I,J)** represent mean ± SD with each dot representing the mean of five adults in **(C)**, five larvae in **(F)**, one larva or 10 adults in **(I)**, and 50–200 flies in **(J)**. Statistical significance was calculated using one-way ANOVA followed by the Šídák’s multiple comparisons test in **(C,F,J)**, two-way ANOVA followed by Tukey’s test in **(G,I)** and the log-rank test and Gehan–Breslow–Wilcoxon test in **(F,H)**. ns, not significant. *p < 0.05.

Our phylogenetic analysis revealed that flies possess two putative TMBIM5 orthologs, CG1287/Mics1 and CG2076/dTmbim5. CG1287/Mics1 is encoded by a single exon and unfortunately only one RNAi line was available to study its function (Figure 4A). Neural (elav-Gal4) as well as ubiquitous KD (tubP-Gal4) did not affect development and lifespan (Figure 4B). Because of the prominent expression of CG1287/Mics1 in testes (Figure 2) and the much stronger expression in male flies (Figure 4C), we also studied male fertility after induction of ubiquitous CG1287/Misc1 KD. Our results show that males with KD of CG1287/Mics1 raised and maintained at 29°C were sterile. In contrast, males raised and maintained at 25°C turned out to be fertile. This is an interesting finding, as we were able to detect only a marginal downregulation in male flies (Figure 4C). Thus, small alterations of CG1287/Mics1 abundance in male testis supposedly have a strong impact on fertility.

To investigate the function of dTmbim5, we could again use two independent RNAi lines. RNAi #1 targets exon 1–4 of dTmbim5 while RNAi #2 targets exon 4. Both isoforms of dTmbim5 that only differ in the 5′ UTR are covered by these two RNAi lines (Figure 4D). Ubiquitous KD with tubP-Gal4 caused lethality at the pupa stage while flies with muscular and neural KD reached adulthood (Figure 4E). Both RNAis resulted in a similarly efficient knockdown in tubP-Gal4 L3 larvae (Figure 4F). Eclosion rates of Mef2-Gal4 KD flies were similar to controls (Figure 4G) but lifespan was reduced with RNAi line #1 being more effective (Figure 4H). Also crawling speed of Mef2 > > dTmbim5-RNAi L3 larvae and climbing ability of young (2-day-old) and old (30-day-old) adults was significantly reduced in RNAi #1 flies (Figure 4I). This was only studied in RNAi #1 flies. Neural KD in both RNAi lines resulted in a lower eclosing rate of pupae (Figure 4J) and a shortened lifespan (Figure 4K). Together these data suggest that dTmbim5 is necessary for fly development and lifespan. Its KD debilitates the function of somatic-muscular and neural tissues. KD of the other TMBIM5 ortholog CG1287/Mics1 in contrast appears to be mainly important for male fertility.

### Ubiquitous Knockdown of dTmbim5 Reduces ATP and Results in a More Pro-apoptotic State

**FIGURE 5:**
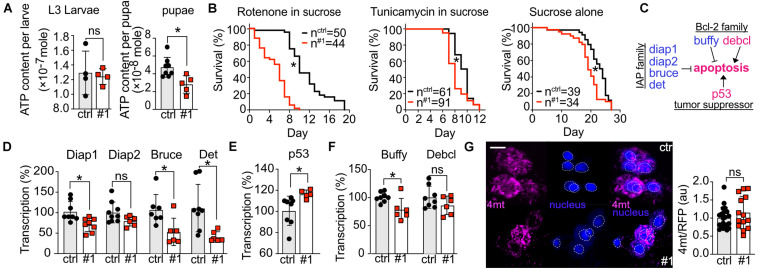
Knockdown of dTmbim5 results in reduced ATP levels and a pro-apoptotic dysregulation of genes involved in apoptosis. **(A)** ATP levels per larva or pupa of tubP > > dTmbim5-RNAi flies. **(B)** Lifespan on food containing rotenone (10 μM in 5% sucrose), tunicamycin (10 μM in 5% sucrose), or sucrose (5%) alone. **(C)** Genes that regulate apoptosis in flies. Blue: anti-apoptotic. Magenta: pro-apoptotic. **(D–F)** Abundance of genes involved in apoptosis quantified by qPCR in the heads of elav > > dTmbim5-RNAi #1 flies. **(D)** Inhibitor of apoptosis (IAP) proteins, **(E)** p53A, and **(F)** Bcl-2 family members. **(G)** Relative mitochondrial calcium levels in Pdf neurons quantitated by 4mtGCaMP3 (4mt) fluorescence normalized to RFP expressed in Pdf > > dTmbim5-RNAi #1 flies. The 4mt signal was normalized to the mean of the control group as well as the RFP signal in the same neuron. Ctrl, UAS-Always-early-RNAi control group. Scale bar in **(G)** 1 μm. Bars in **(A,D,E,F,G)** represent the mean ± SD and each dot corresponds to a group of two animals in **(A)**, 10 heads in **(D–F)**, and one brain in **(G)**. Statistical significance was calculated using the independent *t* test in **(A,D,E,F,G)** and the log-rank test and Gehan–Breslow–Wilcoxon test in **(B)**. ns, not significant. *p < 0.05. Dotted circles represent the regions of interest.

dTmbim5 contains a mitochondrial targeting signal and human TMBIM5 localizes to the inner mitochondrial membrane where it controls cristae structure (Oka et al., 2008) and mitochondrial function including mitochondrial respiration and ATP production (Seitaj et al., 2020). To clarify how loss of dTmbim5 causes its detrimental effect, we thus focused on mitochondria. We quantified the ATP content of whole L3 larvae and pupae of *tubP* > > *dTmbim5-RNAi #1* because this RNAi resulted in a stronger reduction in longevity as compared to RNAi #2 upon neural and muscular KD (Figure 4). We found a significant decrease of ATP levels in pupae but not in larvae (Figure 5A) in line with the arrested development at the pupae stage. Our data suggest that KD of dTmbim5 leads to mitochondrial dysfunction at the pupa stage in flies.

To further explore how loss of dTmbim5 reduces lifespan, we fed dTmbim5 neural KD flies with specific toxins. The mitochondrial complex I inhibitor rotenone causes mitochondrial stress and induces apoptosis (Li et al., 2003; Singh et al., 2007). Compared to control flies, dTmbim5 KD flies displayed a further reduction in lifespan when treated with rotenone (Figure 5B). Flies fed with the ER-stress-inducer tunicamycin, an N-glycosylation inhibitor, however, had a similar reduction in lifespan as those fed sucrose alone (Figure 5B). These results suggest that dTmbim5 KD flies are more vulnerable to mitochondrial stressors than ER stress.

We next quantified the expression levels of genes involved in apoptosis because KO of human TMBIM5 was shown to make cells more susceptible to apoptosis of the intrinsic pathway involving cytochrome *c* release (Oka et al., 2008; Seitaj et al., 2020). Similar to vertebrates, apoptosis in flies is also regulated by the correct balance between pro-apoptotic and anti-apoptotic proteins (Figure 5C; Steller, 2008). We therefore quantified the expression levels of several such genes in the heads of neural KD 2-day-old flies using qPCR to investigate the reasons why those flies (elav > > dTmbim5-RNAi #1) started to die massively on day 4 after eclosion (Figure 4K). In flies there are four so-called Inhibitor of apoptosis (IAP) proteins: Diap1, Diap2, Bruce, and Det (Orme and Meier, 2009). Except for Diap2, the mRNA levels of all of them were reduced by neural KD of dTmbim5 (Figure 5D). p53A is the most abundant p53 isoform in flies, responding to DNA damage and triggering apoptosis (Zhang et al., 2015). Ubiquitous overexpression of p53A kills flies at an early developmental stage (Zhang et al., 2015). KD of dTmbim5 in the nervous system induced p53A expression (Figure 5E). The Bcl-2 family of anti-apoptotic proteins in particular regulates apoptosis by affecting mitochondrial permeability to Cytochrome *c* [reviewed by Singh et al. (2019)] which is facilitated by TMBIM5 KO (Oka et al., 2008; Seitaj et al., 2020). In flies, there are two Bcl-2 members: the anti-apoptotic ER protein Buffy (Quinn et al., 2003; Doumanis et al., 2007) and the pro-apoptotic mitochondrial protein Debcl (Colussi et al., 2000; Doumanis et al., 2007). Transcriptional levels of Buffy were reduced in the head of dTmbim5 neural KD flies, while Debcl remained unchanged (Figure 5F).

Based on the proposed function of mammalian TMBIM6 and the bacterial TMBIM protein BsYetJ as putative Ca^2+^ channels (Bultynck et al., 2014; Lisak et al., 2015), we then examined mitochondrial Ca^2+^ levels of 2-day-old flies by UAS-4mtGCaMP3, UAS-RFP in individual Pdf neurons although dTmbim5 is not very strongly expressed in the heads of young flies. We nevertheless chose this approach because Pdf neurons are easily detected and fluorescence per neuron can be accurately quantified by confocal microscopy (Renn et al., 1999; Manning et al., 2017). Here we observed only a non-significant increase in mitochondrial Ca^2+^ levels in Pdf neurons of Pdf > > dTmbim5-RNAi #1 flies (Figure 5G). Together these results suggest that dTmbim5 KD causes a mitochondrial dysfunction characterized by reduced ATP production and an enhanced vulnerability resulting in a shift toward a more pro-apoptotic state in line with its detrimental effect on the well-being of flies at different stages of development.

### Ubiquitous and Muscular but Not Neural Knockdown of dTmbim6 Results in Developmental Lethality

**FIGURE 6:**
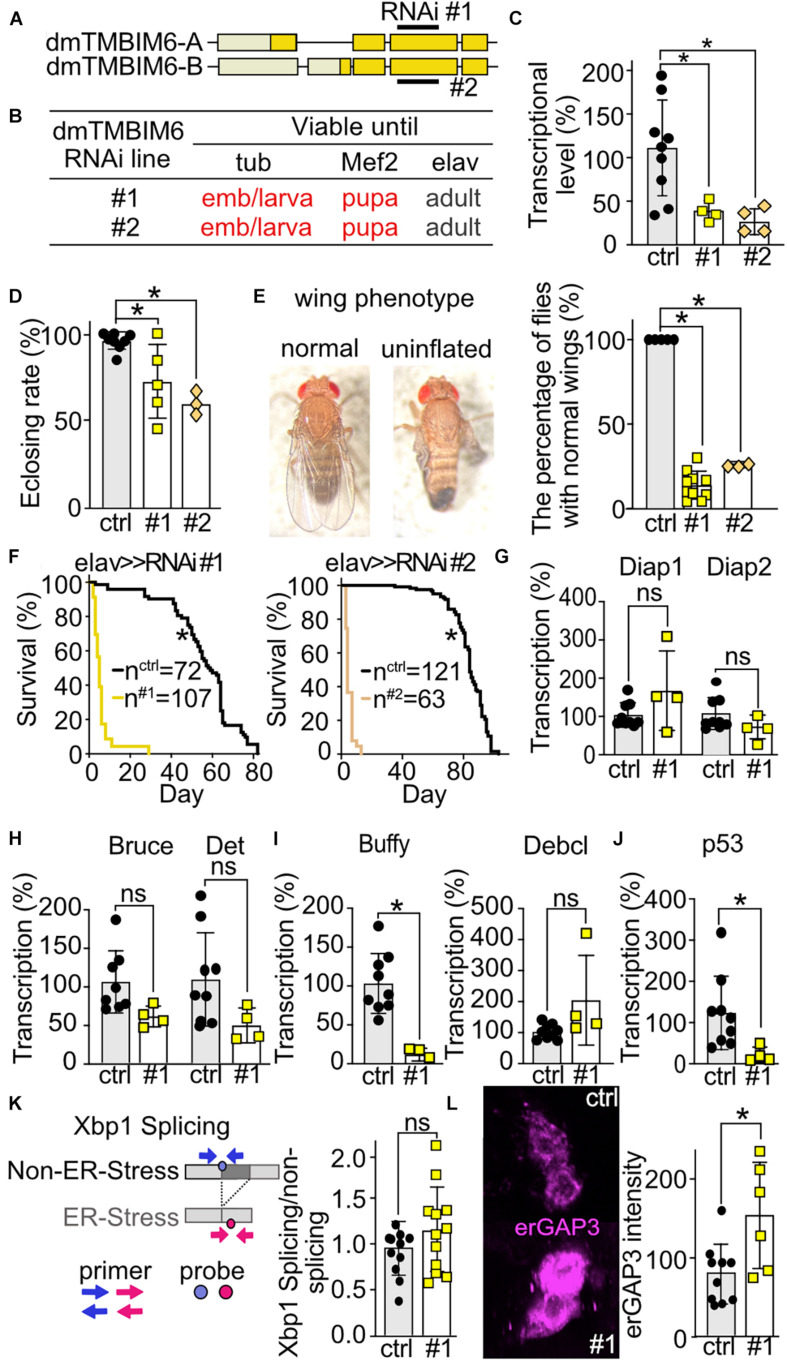
Ubiquitous or muscular knockdown of dTmbim6 leads to developmental lethality while neural knockdown affects wing inflation and dramatically shortens lifespan which correlates with increased ER Ca^2+^ levels but not ER stress. **(A)** Splicing isoforms of dTmbim6. Lighter color represents the UTR and the darker color the open reading frame. The targeting sequence of the two RNAi lines is indicated. **(B)** Viability of dTmbim6 KD flies in two RNAi lines with the indicated Gal4 drivers. Red means death. **(C)** KD efficiency of UAS-dTmbim6-RNAi (#1, #2) assessed by qPCR from whole Mef2 > > dTmbim6-RNAi L3 larvae. **(D)** Eclosion rate of elav > > dTmbim6-RNAi flies. **(E)** Examples of a fly with normal wings and a fly with uninflated wings. Percentage of elav > > dTmbim6-RNAi flies with normal wings. **(F)** Lifespan of elav > > dTmbim6-RNAi flies. mRNA levels of **(G,H)** inhibitor of apoptosis (IAP) proteins, **(I)** Bcl-2 family members and **(J)** p53 of elav > > dTmbim6-RNAi #1 flies. **(K)** ER stress was quantified by detecting spliced and unspliced *Xbp1* using qPCR in the heads of elav > > dTmbim6-RNAi #1 flies. **(L)** ER Ca^2+^ levels quantified by erGAP3 fluorescence in Pdf neurons of Pdf > > dTmbim6-RNAi #1 flies. Ctrl, UAS-Always-early-RNAi control group. Scale bars in **(L)**, 1 μm. Bars in **(C–E,G–L)** represent the mean ± SD and each dot a group of 5–10 animals in **(C)**, 50–200 flies in **(D,F)**, 10 heads in **(G–K)**, and one brain in **(L)**. Statistical significance was calculated using One-way ANOVA followed by the Dunnett’s test **(C,D,E)**, the log-rank test and Gehan–Breslow–Wilcoxon test in **(F)**, and the independent *t* test in **(G–L)**. ns, not significant *p < 0.05.

To explore the phenotype of dTmbim6, we used two independent RNAi lines of the same long hairpin RNA expression construct. Both constructs obviously target the same sequence in the third exon of dTmbim6, but are randomly inserted into the genome (Figure 6A). No off-target was identified for the targeting sequence (Dietzl et al., 2007). We used both lines to exclude possible effects on phenotype by the insertion of the element itself. Ubiquitous and muscular-specific KD by both RNAis caused lethality at very early developmental stages – the majority of KD offspring died at the embryonic stage and very few reached the L1 larval stage (Figure 6B). In contrast, neural-specific KD did not cause developmental lethality and long hairpin RNA-expressing flies reached adulthood (Figure 6B). To prove the efficiency of KD, we isolated mRNA from L3 larvae of dTmbim6 muscle-specific KD flies. As dTmbim6 expression is high in muscles, we expected an efficient RNAi-mediated silencing of dTmbim6 KD by Mef2-Gal4 driver. Indeed, both RNAi lines significantly reduced dTmbim6 mRNA levels in L3 larvae (Figure 6C). We further examined the importance of dTmbim6 in the nervous system. Compared to control, neural KD of dTmbim6 significantly reduced the eclosion rate, independent of the RNAi line used (Figure 6D). Interestingly, roughly 80% of flies with neural KD of dTmbim6 were not able to inflate their wings (Figure 6E). Wing inflation normally occurs shortly after eclosion and failure of wing inflation frequently coincides with neuronal dysfunction (Vanden Broeck et al., 2013; Cagin et al., 2015). We also observed a dramatically reduced lifespan with both RNAis (Figure 6F). These results imply that dTmbim6 plays a major role during development.

### Knockdown of dTmbim6 Increases ER Calcium Levels but Does Not Affect *Xbp1* Splicing

To clarify the cause of dTmbim6 KD-induced premature death, we assessed the expression of apoptotic genes in heads of flies with neural KD of dTmbim6 (elav > > dTmbim6-RNAi #1) 2 days post eclosion, just before these flies started to die (Figure 6F). We again used only one RNAi line for these more mechanistic studies because both lines were almost indistinguishable. Unlike dTmbim5, KD of dTmbim6 had no effect on the mRNA levels of the IAP genes (Figures 6G,H). However, the mRNA encoding the anti-apoptotic Bcl-2 protein Buffy was strongly down-regulated (Figure 6I), while the expression of the pro-apoptotic Debcl was unchanged (Figure 6I). Interestingly, p53A was significantly reduced compared to control (Figure 6J). Based on reports that suggested an inhibitory role for TMBIM6 against the ER stress sensor protein inositol-requiring transmembrane kinase/endoribonuclease 1α (IRE1α) in mammalian cells (Lisbona et al., 2009), we next studied ER stress levels in these flies. Activation of IRE1α results in alternative splicing of the X-box–binding protein 1 (Xbp1) mRNA. Only spliced mRNA translates into the active form of the transcription factor (Yoshida et al., 2001). This process is conserved in the fly (Plongthongkum et al., 2007). We therefore quantitated the amount of spliced and unspliced *Xbp1* (CG9415) using qPCR (Figure 6K) which yielded no differences (Figure 6K). ER Ca^2+^ levels were, however, increased in the Pdf neurons of Pdf > > dTmbim6-RNAi flies (Figure 6L) similar to observations in mice (Lisak et al., 2016; Philippaert et al., 2020). In contrast to dTmbim5, dTmbim6 is strongly expressed in the heads of young adult flies (Figure 2A). In summary, these data suggest that KD of dTmbim6 shifts cell fate toward death by greatly reducing the expression of the Bcl-2 protein Buffy and an increased ER Ca^2+^ load. These data suggest that ER stress is not directly involved in the detrimental phenotype of dTmbim6 KD.

## Discussion

In this study, we identified seven TMBIM family members in the fruit fly *Drosophila melanogaster* and further compared and investigated five members in detail: CG3798/Nmda1, CG3814/Lfg, CG1278/Mics1, CG2076/dTmbim5, and CG7188/dTmbim6. By ubiquitous and tissue-specific RNAi-mediated knockdown, we show that dTmbim5 and dTmbim6, but not CG3798/Nmda1 and CG3814/Lfg, are indispensable for the development and adult health of flies. Despite their shared protein structure, lethality after KD of the different TMBIM family members seems to occur through different mechanisms.

To rule out off-target effects, we used two independent RNAi lines for each TMBIM gene where available. The two RNAi-lines used to silence CG3798/Nmda1 target different sequences with a small overlap. For CG3814/Lfg, one RNAi targets the last exon and one the 3′ UTR. For CG1278/Mics1, the RNAi line targets the ORF of the single transcript. For dTmbim5, the two RNAi lines target different regions. Both showed the same knockdown efficiency and resulted in the same phenotype, including lethality at the pupal stage after ubiquitous KD (tubP-Gal4). Pan-neural KD was semi-lethal with a low eclosing rate; surviving flies displayed a reduced lifespan compared to controls. For dTmbim6, two RNAi lines expressing the same long hairpin RNA (dna1660) but randomly inserted on the 2nd chromosome had the same efficiency. Both RNAis resulted in embryonic/larval lethality by tubP-Gal4 and pupal lethality by Mef2-Gal4, as well as identical phenotypes by elav-Gal4, such as an abnormal wing phenotype, disturbed eclosion and a shortened lifespan. In addition, no off-target effects associated with these RNAi lines have been identified in previous work (Dietzl et al., 2007). In summary, these data suggest that the phenotypes reported here are not caused by off-target effects of the used RNAi lines.

The possible ortholog of CG3814/Lfg, TMBIM2 localizes to the membrane of Golgi apparatus (Lisak et al., 2015) and is ubiquitously expressed with a remarkable high expression in the hippocampus (Somia et al., 1999). TMBIM2 KO mice are viable but suffer from cerebellar atrophy (Hurtado de Mendoza et al., 2011). KD of CG3814/Lfg by RNAi #1 also used in this study driven by ddc-Gal4 (cells expressing dopa decarboxylase) led to a disabled climbing ability and a shortened lifespan (M’Angale and Staveley, 2016). In this study, we did not assess effects on lifespan of CG3814/Lfg KD.

The possible ortholog of CG3798/Nmda1, mammalian TMBIM3, is a transmembrane protein that localizes to the Golgi apparatus and the ER. Mouse TMBIM3 is mainly expressed in the central nervous system (Nielsen et al., 2011) and its deficiency does not result in an obvious phenotype (Nielsen et al., 2011). *TMBIM3* knockout increases susceptibility against ER stress induced by tunicamycin or thapsigargin (Rojas-Rivera et al., 2012; Habib et al., 2019). Previous work in flies found that KD of CG3798/Nmda1 (named TMBIM3 in this publication) only resulted in apoptosis in larvae and a reduced eclosing rate in the presence of tunicamycin (Rojas-Rivera et al., 2012). A CG3798/Nmda1 and dTmbim6 double KD displayed a stronger reduction of eclosion (Rojas-Rivera et al., 2012). In our hands, KD of dTmbim6 alone was detrimental. As CG3798/Nmda1 and dTmbim6 are only remotely related to each other (Figure 1), we think that the observed effect might also be caused by an additive effect and not because the two proteins act synergistically. CG3798/Nmda1 and CG3814/Lfg, in contrast, are two highly homologous proteins which are 71% identical and 86% similar and have a partially overlapping expression pattern (Figure 2A). Both genes are located next to each other on the 2nd chromosome and were most probably spawned from a recent gene duplication and might represent paralogs (Figure 3A). One hypothesis could be that the KD of CG3798/Nmda1 KD could be compensated by CG3814/Lfg and vice versa. The expression profile of the two mRNAs does, however, only overlap in the digestive system (Figure 2) which does not support this hypothesis. Alternatively, both gene products (Nmda1 and Lfg, respectively) might have adopted independent functions that are not necessary for the correct development of adult flies. In ongoing analyses, neural (elav-Gal4) double KD of CG3798/Nmda1 (RNAi #1) and CG3814/Lfg (RNAi #1) had no obvious impact on development. Vital flies were obtained and did not display any apparent phenotype suggesting that both gene products have no essential function in neurons. Further research is needed to rule out the functional consequence of Nmda1 and/or Lfg KD in other tissues.

Human TMBIM5 is a mitochondrial inner membrane protein with two potential orthologs in the fly. We consider CG2076/dTmbim5 to be the functional ortholog of TMBIM5 based on the following findings: (i) our phylogenetic analysis that revealed a slightly stronger homology of dTmbim5 with TMBIM5 than CG1278/Mics1 with TMBIM5, (ii) a similar genomic organization of *dTmbim5* and *TMBIM5*, (iii) a similar rather ubiquitous tissue expression of dTmbim5 and TMBIM5, and (iv) the stronger KD phenotype with ubiquitous KD of dTmbim5 resulting in arrested development at the pupa stage while KD of CG1278/Mics1 only resulted in male sterility at 29°C. A TMBIM5-deficient vertebrate model has not yet been described, but in human cell lines loss of human TMBIM5 was shown to result in a disrupted mitochondrial cristae structure and an increased release of cytochrome *c* from mitochondria (Oka et al., 2008) possibly by interfering with the mitochondrial protein synthesis machinery (Seitaj et al., 2020). Although cytochrome *c* release from mitochondria into the cytoplasm is not necessary for activation of apoptosis in flies (Dorstyn et al., 2002), one of the two cytochrome c genes cyt-c-d is required for caspase activation during apoptosis (Arama et al., 2003, 2006) in *Drosophila*. It is yet unclear whether loss of fly dTmbim5 also facilitates cytochrome *c* release. We also found p53A strongly upregulated in dTmbim5 KD flies. In mice, p53 suppresses Bcl-xl, an anti-apoptotic Bcl-2 family protein highly homologous to Bcl-2 (Sugars et al., 2001; Quinn et al., 2003; Doumanis et al., 2007). Unfortunately, Pdf neurons, which are ideal to quantitate organellar Ca^2+^ levels (Renn et al., 1999; Liang et al., 2016; Manning et al., 2017) do not express significant amounts of dTmbim5 during early adulthood (Figures 2A,B). We can therefore not exclude an effect of dTmbim5 KD on mitochondrial Ca^2+^ levels in general.

dTmbim6 is an ER membrane protein expressed in all developmental stages and in all tissues of *Drosophila melanogaster*. Accordingly, its ubiquitous, but also muscle-specific KD was not reconcilable with life (Figure 6B). This is in contrast to previous work where ubiquitous KD using RNAi v3235 driven by the tubP promoter, thus exactly the same setup used in our study, had no effect despite an mRNA reduction of 80% in whole larvae (Rojas-Rivera et al., 2012). We used v3235 and v37108, which have the same targeting sequence (dna1660, 320 bp), but are inserted randomly into the fly genome, and found that ubiquitous KD by both RNAi lines driven by tubP-Gal4 caused lethality at the embryonic/early larva stage while KD using Mef2-Gal4 arrested development at the pupa stage. We therefore used whole L3 wandering larvae by Mef2-Gal4 to quantify mRNA, which demonstrated a reduction of dTmbim6 by around 70% for both lines which is similar to the previous study (Rojas-Rivera et al., 2012). We found that in both RNAi lines, neural-specific KD shortened lifespan and resulted in a strong wing phenotype. These results suggest that TMBIM6, at least in the fly, has important housekeeping functions, which cannot be compensated by other TMBIM proteins. In mice, however, ubiquitous knockout is compatible with life (Chae et al., 2004; Lisak et al., 2016). Regarding the role of TMBIM6 in ER stress and Ca^2+^ homeostasis, our results (obtained in flies) are in line with previous findings obtained in a BI-1 knockout mouse. In these mice, we found increased ER Ca^2+^ levels in phenotypically different cells, lymphocytes, but no hint of constitutive ER stress (Lisak et al., 2016). The same was true in mouse pancreatic β cells which displayed profound changes in glucose-mediated Ca^2+^ regulation and increased levels of IRE1α levels but no differences in downstream effects of IRE1α like increased *Xbp1* mRNA splicing or Ire1-dependent decay of insulin mRNA (Philippaert et al., 2020). This does of course not rule out an increased susceptibility of TMBIM6-deficient cells to ER stress as shown previously in mice (Chae et al., 2004) and in flies, where a reduced eclosing rate was observed in dTmbim6 KD flies subjected to additional ER stress induced by tunicamycin (Rojas-Rivera et al., 2012).

In summary, our study proves the importance of the TMBIM family members in many different organs and tissues of the fly. The fact that the fly possesses essentially the same set of TMBIM proteins as mammals advocates that these proteins have similar functions and imply that some of our findings might also be of importance for humans.

## Data Availability Statement

The original contributions presented in the study are included in the article/supplementary material, further inquiries can be directed to the corresponding author.

## Author Contributions

LZ, AV, and AM: conceptualization and writing. LZ and SB: acquisition of data. LZ, SB, and AM: analysis. All authors contributed to the article and approved the submitted version.

## Conflict of Interest

The authors declare that the research was conducted in the absence of any commercial or financial relationships that could be construed as a potential conflict of interest.

## Publisher’s Note

All claims expressed in this article are solely those of the authors and do not necessarily represent those of their affiliated organizations, or those of the publisher, the editors and the reviewers. Any product that may be evaluated in this article, or claim that may be made by its manufacturer, is not guaranteed or endorsed by the publisher.

## Footnotes

1
www.blast.ncbi.nlm.nih.gov
